# Transcranial direct current stimulation (tDCS) for treatment of major depression during pregnancy: study protocol for a pilot randomized controlled trial

**DOI:** 10.1186/1745-6215-15-366

**Published:** 2014-09-18

**Authors:** Simone Vigod, Cindy-Lee Dennis, Zafiris Daskalakis, Kellie Murphy, Joel Ray, Tim Oberlander, Sarah Somerton, Neesha Hussain-Shamsy, Daniel Blumberger

**Affiliations:** Women’s College Hospital and Research Institute, 76 Grenville Street, Toronto, ON M5S 1B1 Canada; University of Toronto, 563 Spadina Crescent, Toronto, ON M5S 2 J7 Canada; Centre for Addiction and Mental Health, 250 College Street, Toronto, ON M5T 1R8 Canada; Mount Sinai Hospital, 600 University Avenue, Toronto, ON M5G 1X5 Canada; St Michael’s Hospital, 30 Bond Street, Toronto, ON M5B 1 W8 Canada; University of British Columbia, 2329 West Mall, Vancouver, BC V6T 1Z4 Canada

**Keywords:** depression, pregnancy, transcranial direct brain stimulation (tDCS), women

## Abstract

**Background:**

Women with depression in pregnancy are faced with difficult treatment decisions. Untreated, antenatal depression has serious negative implications for mothers and children. While antidepressant drug treatment is likely to improve depressive symptoms, it crosses the placenta and may pose risks to the unborn child. Transcranial direct current stimulation is a focal brain stimulation treatment that improves depressive symptoms within 3 weeks of treatment by inducing changes to brain areas involved in depression, without impacting any other brain areas, and without inducing changes to heart rate, blood pressure or core body temperature. The localized nature of transcranial direct current stimulation makes it an ideal therapeutic approach for treating depression during pregnancy, although it has never previously been evaluated in this population.

**Methods/design:**

We describe a pilot randomized controlled trial of transcranial direct current stimulation among women with depression in pregnancy to assess the feasibility of a larger, multicentre efficacy study. Women over 18 years of age and between 14 and 32 weeks gestation can be enrolled in the study provided they meet diagnostic criteria for a major depressive episode of at least moderate severity and have been offered but refused antidepressant medication. Participants are randomized to receive active transcranial direct current stimulation or a sham condition that is administered in 15 30-minute treatments over three weeks. Women sit upright during treatment and receive obstetrical monitoring prior to, during and after each treatment session. Depressive symptoms, treatment acceptability, and pregnancy outcomes are assessed at baseline (prior to randomization), at the end of each treatment week, every four weeks post-treatment until delivery, and at 4 and 12 weeks postpartum.

**Discussion:**

Transcranial direct current stimulation is a novel therapeutic option for treating depression during pregnancy. This protocol allows for assessment of the feasibility of, acceptability of and adherence with a clinical trial protocol to administer this treatment to pregnant women with moderate to severe depression. Results from this pilot study will guide the development of a larger multicentre trial to definitively test the efficacy and safety of transcranial direct current stimulation for pregnant women with depression.

**Trial registration:**

Clinical Trials Gov NCT02116127.

## Background

Depression is the second leading cause of disability in women and is the most common morbidity in pregnancy [1, 2]. Left untreated, *in-utero* exposure to depression can lead to serious impacts on the child, including preterm birth, reduced birth weight, small head circumference and lower Apgar scores during the neonatal phase, and increased risk of poor developmental and emotional outcomes, including higher risk of both internalizing (for example, anxiety) and externalizing (for example, attention deficit hyperactivity disorder) disorders in childhood [3, 4]. Given the high prevalence and substantial impact on children, rapid, effect treatment of depression during pregnancy is a high priority.

Unfortunately, no existing treatment option for depression in pregnancy is without potential risk. There are two standard types of treatment for depression in pregnancy: (1) psychotherapy and (2) antidepressant medication. Mild depression during pregnancy can be treated with psychotherapy, but this is unlikely to result in substantive improvements if a woman has moderate to severe depression (effect sizes ranging from 0.15 for interpersonal therapy to 0.39 for cognitive behavioural therapy) [5]. It may also take several weeks to months to improve depressive symptoms, leaving the mother and fetus exposed to the effects of untreated (or incompletely treated) depression during that time. Antidepressant medication is an effective treatment option (effect size approximately 0.61 for selective serotonin reuptake inhibitors compared with placebo) and is the recommended first line of treatment for major depression in pregnancy [5, 6]. However, concerns about the safety of antidepressant medication exposure for the developing fetus limit its acceptability among patients and providers. Its use has been associated with small absolute risk increases of neonatal cardiovascular malformations and neonatal pulmonary hypertension, as well as reports of spontaneous abortion, low birth weight, preterm birth, fetal death and seizures among exposed infants [7–21]. Antidepressant medication also crosses the placenta and uncertainty remains about whether there are any long-term negative effects on the child [22].

Transcranial direct current stimulation (tDCS) is a novel brain stimulation technique that has the potential to be an ideal treatment option for depression in pregnancy. It is a localized brain stimulation treatment that is based on an understanding that there are abnormalities in activity in frontal brain regions in this disorder, particularly the left and right dorsolateral prefrontal cortices [23–26]. This technique involves the application of a small current (1 to 2 mA) between two electrodes placed on the scalp that induces localized neuronal activity in the prefrontal cortex [27, 28]. A meta-analysis of ten studies reported that compared with sham tDCS, active tDCS was more effective in reducing symptoms of depression after 2 to 3 weeks of treatment [29]. A recent large, randomized sham-controlled trial confirmed these findings with an effect size on depression outcome of 0.49 [30]. Importantly, tDCS has been shown to be safe in a number of trials [31–37]. Worldwide, no major adverse events have been reported in 2,000 to 3,000 known human subjects [38]. The procedure produces a mild tingling sensation initially, which usually resolves within 30 seconds. In a study examining the side effects of 567 sessions of tDCS with 77 healthy controls and 25 patients, the most common side effects of tDCS were a mild tingling sensation, moderate fatigue and a light itching sensation under the stimulation electrodes. After tDCS, headache (10%), nausea (<3%) and insomnia (<1%) have been reported [39]. Relevant to the application of this method in pregnant women, a recent brain imaging study demonstrated that tDCS can produce changes in regional brain activity in the prefrontal cortex without inducing changes in any other brain area [28]. Furthermore, another recent tDCS study found no changes in autonomic function, ventilation rate or core body temperature attributable to tDCS applied in healthy volunteers [40]. Thus, the localized stimulation presents no theoretical risk to the fetus. Furthermore, it is an accessible treatment option. The equipment required is inexpensive and portable and can be administered by trained technicians or by patients themselves at home once the stimulation parameters are appropriately programmed.

In summary, tDCS is a novel technique with strong promise to allow women presenting with depression during pregnancy a safe, nonpharmacological treatment. Herein we present the methodology of a pilot randomized controlled study that evaluates the feasibility of a trial protocol for tDCS in pregnant women with depression (research protocol version 3; 10 April 2014). The primary objective of this protocol is to assess feasibility, acceptability and adherence with a prospective, two-armed, sham-controlled, pilot randomized controlled trial (RCT) protocol to evaluate tDCS for the treatment of depression in pregnant women. This will guide the development of a larger multisite RCT to definitively evaluate the efficacy of tDCS treatment for depression during pregnancy. The secondary objective of the pilot study is to measure the effect of tDCS on reducing depressive symptoms immediately post-treatment among women who have moderate to severe depression in pregnancy. This will generate a preliminary effect size to inform the sample size required for a more definitive RCT.

## Methods/design

### Study design, setting and recruitment

**Figure 1:**
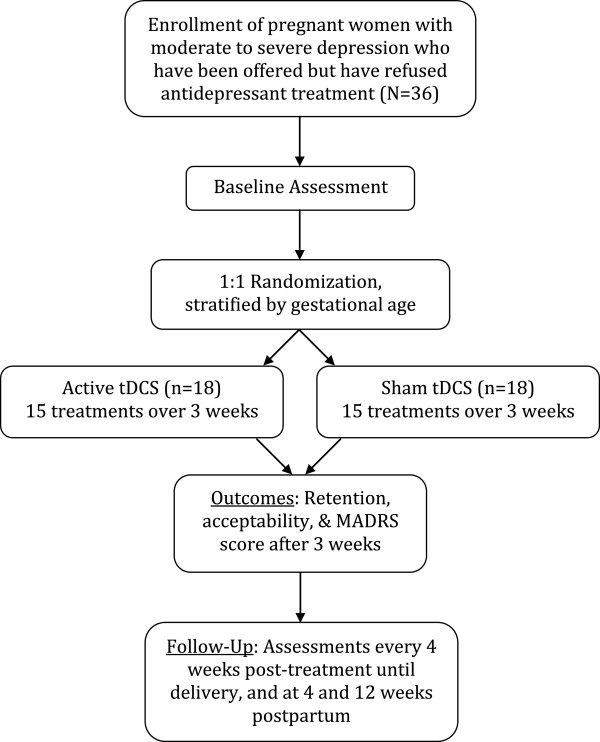
**Trial design.** Randomization schema of a pilot randomized controlled trial on the feasibility of transcranial direct current stimulation to treat depression during pregnancy. MADRS, Montgomery Asberg Depression Rating Scale; tDCS, transcranial direct brain stimulation.

This is a two-armed, sham-controlled, pilot RCT (see Figure 1). The pilot RCT is recruiting women with depression in pregnancy over the course of 1 to 2 years from specialty perinatal mental health programmes in the Departments of Psychiatry and Obstetrics and Gynecology at two university-affiliated hospitals in Toronto, Ontario, Canada (Mount Sinai Hospital and Women’s College Hospital). Both hospitals receive referrals for mental health care of women during pregnancy from all over the Greater Toronto Area (population ≈ 3.5 million). To help achieve adequate participant enrolment, flyers have been placed at the recruitment sites, and physicians providing antenatal care at both sites have been informed of the study to assist with identification of potentially eligible participants. Physician education about the study has included small and large group presentations, as well as an information sheet. Both the intervention and sham control are delivered in the Clinical Research Unit at Mount Sinai Hospital by trained obstetrical research nurses to ensure appropriate obstetrical monitoring during the procedures. The active phase of the study involves a total of 15 sessions of the intervention or sham control lasting 30 minutes each over approximately 3 weeks (one per weekday). Participants are then followed through the remainder of the pregnancy, and until 12 weeks postpartum with outcome data collected by Masters-level trained research staff. Outcome data collectors, participants and physicians providing clinical care are blind to study group allocation. The study was approved by the research ethics boards of Women’s College Hospital (Reference Number 2013-0085-B) and Mount Sinai Hospital (Reference Number 13-0320-A) in Toronto, Ontario. The study is registered with ClinicalTrials.Gov under the identifier NCT02116127. The research ethics boards of both institutions involved in the study are notified if any changes are made to the study protocol. The trial registration is also updated as appropriate.

### Eligibility criteria

To be included in the study, women must: (1) be pregnant and aged at least 18 years; (2) be >14 weeks and ≤32 weeks of gestation at enrolment; (3) meet diagnostic criteria for major depressive disorder, with a current major depressive episode of at least moderate severity (according to version 5 of the Diagnostic and Statistical Manual of Mental Disorders, DSM-5) confirmed by an assessment with the Mini International Neuropsychiatric Interview [41]; (4) have been offered, but declined to use, antidepressant medication by their treating clinician at one of the two recruitment sites; (5) be assessed by the principal investigator to be eligible for outpatient psychiatric treatment; and (6) be capable of consenting to treatment and understanding study explanations and questionnaires in English. Our sample is restricted to women between 14 and 32 weeks gestation to increase the likelihood that participants remain pregnant through the active phase of the study (that is, women in the first trimester of pregnancy have a greater chance of spontaneous abortion) [42]; women >32 weeks gestation would have a higher likelihood of delivery during the intervention phase, especially given that women with depressive symptoms have an increased risk of late preterm births [43]. We are only enrolling women who have been offered, but declined, treatment with antidepressant drugs because this treatment, despite its potential disadvantages, remains the standard of care in this population [5]. Participants are excluded from the study if they: (1) have a DSM-5 diagnosis of alcohol or substance use or dependence in the previous six months; (2) have concomitant major and unstable medical or neurological illness or history of seizure; (3) are currently taking carbamazepine (as this may reduce the efficacy of tDCS treatment); (4) are currently taking benzodiazepines on a daily basis (not including intermittent use of lorazepam of <2 mg per day); (5) have metal implants in the cranium, electrical implants in the body, or nonintact skin on the scalp areas where stimulation electrodes will be placed (as per tDCS device manufacturer specifications); (6) have major obstetrical complications or a known fetal anomaly during the current pregnancy as determined by the investigator team; (7) have a history of very preterm (that is <32 weeks gestation) delivery for a previous pregnancy; or (8) are planning to leave the study city prior to delivery during the current pregnancy.

### Informed consent procedures

Potentially eligible women who are willing to hear more about the study receive a detailed study explanation from the study research coordinator. Subjects are provided with a clear explanation of the objectives, procedures, risks and benefits of the study and all questions are answered. Questions are asked of subjects to ensure that they understand the nature of the research, risks and potential benefits of study participation, and their rights as research subjects prior to their signing the informed consent document. Interested patients are asked to sign the informed consent form before entry into the study. Informed consent is obtained before any study assessments and study procedures are performed and before any private information is recorded. Participants are given meaningful opportunities during the study to provide ongoing consent to continuation with the study protocol. Among pregnant women who are faced with treatment decisions, it has been shown that the opinions of partners, and even family and friends can play a large role [44]. We routinely request that a woman invite her partner to be present for the initial discussions about the study, so that all of the individuals involved in the decision about whether or not to enrol in the study have adequate information about it.

### Allocation of interventions

After informed consent procedures and collection of baseline data, eligible and consenting women are randomized to receive either the active tDCS intervention or a sham control condition. A research assistant external to the study generated the allocation sequence using a random permuted block randomization table. Randomization identification numbers (RIDs) in the table are stratified based on gestational age at enrolment, to increase the likelihood that groups will be balanced with respect to gestational age at entry. As such, the first stratum of RIDs is assigned to participants who are less than 24 weeks pregnant at enrolment. The second stratum of RIDs is assigned to participants who are 24 or more weeks pregnant at enrolment. The group allocations were placed in sealed envelopes with RIDs written on the front by the external research assistant. The study research coordinator assigns RIDs sequentially, then selects a presealed envelope with the RID written on the front to include in the participant’s research chart. At the time of the session, the obstetrical research nurse who administers the tDCS intervention or sham opens the envelope to determine the group allocation. It is necessary for the research nurse to know the group allocation because the tDCS device must be programmed differently depending on whether the participant is to receive the active tDCS intervention or control. The research nurse does not provide clinical care nor collect outcome data.

### Interventions

A total of 15 sessions lasting 30 minutes each are administered to participants over approximately 3 weeks (one per weekday) by a trained obstetrical research nurse at the hospital. Training in the administration of active tDCS and sham procedures was provided by DB (the coprincipal investigator) who has experience in training tDCS technicians and has conducted previous tDCS treatment trials using trained technicians to administer the intervention [45–47]. Participants are awake and sit as upright as possible during treatment. The tDCS obstetrical research nurse who administers the intervention measures maternal blood pressure and heart rate prior to the procedure. Participants receive continuous fetal monitoring for 10 minutes prior to the tDCS or sham stimulation, for the duration of the session and for 10 minutes afterward using a nonstress test. For participants who are too early in their pregnancy to receive continuous fetal monitoring (that is, prior to 24 weeks gestation), a fetal heart rate measurement is recorded 10 minutes before and after each session. Maternal blood pressure and heart rate are recorded after each session for all participants. The active tDCS intervention and sham protocols are based on those provided to nonpregnant research participants in the investigators’ previous tDCS trials [45–47]. The active tDCS intervention is active 2 mA tDCS. Direct current is transferred continuously for 30 minutes with a pair of saline-soaked sponge electrodes (contact area of 5 × 7 cm), and delivered by a specially developed, battery-driven constant current stimulator. The electrodes are placed over F3 and F4 according to the 10–20 international system for electroencephalogram placement. This has been shown to be a relatively accurate method of dorsolateral prefrontal cortex localization by neuronavigation methods [48]. It has been used before in tDCS studies targeting the dorsolateral prefrontal cortex (an important area implicated in the etiology of depression) and has demonstrated efficacy in treatment studies of resistant depression [34, 35, 47]. The control or ‘sham’ stimulation is administered using the same stimulation parameters and at the site of active treatment, but the current is turned off after 30 seconds. Typically, tDCS induces an itching or tingling sensation for the first 30 seconds of its application, which then fades, making this an appropriate blinding method. During the sessions themselves, the obstetrical research nurses have been instructed to limit engagement with participants, in order to prevent co-intervention and to prevent accidental unblinding of participants during the session.

Women continue to receive clinical care from their respective clinical programmes during the trial, however, their clinical care providers are blind to their group allocation. Although this care may include psychotherapeutic intervention that is initiated prior to completion of the active tDCS treatment phase (if the clinic psychotherapy waiting list is short), significant improvement within the first 3 weeks of psychotherapeutic treatment is unlikely. As such, this is an ideal opportunity to evaluate the efficacy of a new treatment, without depriving women of nonpharmacological standard care. For ethical reasons, clinical care might include offering of antidepressant medication as indicated because, despite its risks, antidepressant medication remains the standard of care for moderate to severe depression. If a participant begins antidepressant medication during the course of the study, this will be documented but the participant will remain in the study. Specific criteria for termination of participation are active suicidal ideation, psychosis, treatment-emergent mania and acute pregnancy complications during and after treatment (as determined by the study team). If any termination criteria are met, the participant will end all sessions, independent of group allocation, and be followed-up by her physician. Participants who terminate early will be included in the intention-to-treat analysis, and we will continue to collect follow-up data if participants are willing.

### Study schedule

The study schedule is described in Table 1. A baseline visit with the research coordinator (blind to group allocation) occurs in person, prior to the start of the active study phase to collect baseline demographic and health service use information, and to collect baseline measures on mental health symptom scales. During the active study phase, study session visits are daily on weekdays over 3 weeks when participants are receiving either the tDCS intervention or the sham treatment. The obstetrical research nurse administering the session records information on each visit, including maternal and fetal heart rate, maternal blood pressure, start and stop time of active or sham tDCS, and any interruptions, using a session log. In addition, active study phase interviews are conducted in person with the research coordinator following the final session each week. Follow-up questionnaires are then completed in person or by telephone (depending on patients’ preferences) every 4 weeks until delivery, and at 4 and 12 weeks postpartum. Several post-recruitment retention strategies are used to retain participants in this study and optimize follow-up data collection rates. First, women receive transit tokens or parking reimbursement as required. Second, subsequent to the active study phase, women are contacted by telephone and email to remind them about their follow-up assessments. Women also receive email updates about recruitment targets and follow-up completeness to help them understand the contribution they are making by remaining in the study.

**Table 1:** **Study schedule for the participants in the pilot randomized controlled trial of transcranial direct current stimulation (tDCS) for treatment of major depression during pregnancy**

Measure	Study period									Purpose
	Prior to active study phase		Active study phase				Follow-up			
	Enrolment	Baseline visit	Daily	End of week 1	End of week 2	End of week 3	Every 4 weeks until delivery	4 weeks postpartum	12 weeks postpartum	
Eligibility screen	X									Feasibility
Informed consent	X									Feasibility
Group allocation		X								Feasibility
Demographic questionnaire		X								Covariates
Mini International Neuropsychiatric Interview		X								Covariates
Health service use questionnaire		X		X	X	X	X	X	X	Covariates
Montgomery Asberg Depression Rating Scale		X		X	X	X	X	X	X	Adherence, efficacy
Edinburgh Postnatal Depression Scale		X		X	X	X	X	X	X	Adherence, efficacy
Pregnancy Experience Scale		X		X	X	X	X	X	X	Adherence, efficacy
State-Trait Anxiety Inventory		X		X	X	X	X	X	X	Adherence, efficacy
Pregnancy complications		X		X	X	X	X	X		Acceptability, adherence
Fetal monitoring			X							Acceptability, adherence
Treatment allocation questionnaire				X	X	X				Feasibility
Toronto Side Effects Questionnaire				X	X	X				Acceptability, adherence
Treatment perceptions interview						X				Acceptability, adherence
Neonatal outcomes								X		Acceptability, adherence
Infant characteristics questionnaire									X	Acceptability, adherence
Ages and Stages Questionnaire-3									X	Acceptability, adherence

### Outcomes

The primary outcome measures for the pilot study are (1) feasibility, (2) acceptability and (3) adherence with the trial protocol. Feasibility describes how well the trial protocol can be implemented. We record feasibility data related to (a) eligibility (for example, proportion of reproductive mental health clinic patients eligible), (b) recruitment (for example, number, nonparticipation reasons) and (c) timing (for example, time before participant begins treatment). We also assess the feasibility of our blinding methods by asking women whether they believe they are receiving active or sham stimulation at the end of each week of the active study phase. Acceptability refers to women’s satisfaction with and perceptions of the intervention and what side effects or adverse outcomes (if any) are experienced. To demonstrate acceptability, a focus on fetal, maternal, neonatal and child outcomes is essential. Acceptability measures are: (a) fetal monitoring during each session (as described above); (b) the Toronto Side Effects Scale at the end of each week of the active study phase [49]; (c) a semistructured treatment perceptions interview at the end of the intervention phase; and (d) questionnaires to assess additional maternal, neonatal infant and child outcomes. We do not expect any specific maternal or neonatal complications to occur as a result of this protocol. Therefore, questionnaires to assess maternal and neonatal outcomes ask specifically about the perinatal health indicators recommended by the Canadian Perinatal Surveillance System [50]. These questionnaires are administered at the end of each week of the active study phase, and at each follow-up visit until the 4-week postpartum visit. To increase accuracy of the self-report measures for these perinatal health indicators, we provide participants with a copy of the relevant questionnaires. We also request that women allow us to obtain their infant outcome data from their clinical records to validate the self-report data against that for the pilot. For early childhood outcomes, temperament is assessed using the Infant Characteristics Questionnaire, an instrument that uses a series of similarly constructed questionnaires developed to assess parental perceptions of difficult infant temperament [51]; and infant development is assessed using the Ages and Stages Questionnaire, a 30-item instrument that uses a series of similarly constructed questionnaires to screen child development from 1 through 60 months of age [52, 53]. Finally, we assess adherence, defined as the degree to which the trial protocol is followed. Adherence-related measures include (a) the number of women who complete the 15 active study phase sessions and reasons for discontinuation, (b) the total number of sessions attended overall, and within the intended active study phase (that is, 3 weeks) and (c) the rate of follow-up data collection. Follow-up data collection is especially important because ideally, in the large multicentre trial, we would like to be able to obtain detailed measures of neonatal health in the postpartum period and follow children longitudinally to measure child outcomes.

Secondary efficacy outcomes are measured using questionnaires administered by the research coordinator at baseline, weekly during treatment, immediately post-treatment at the end of the active study phase (primary efficacy measure), at 4-week intervals until delivery, then at 4 and 12 weeks postpartum, to obtain information about the duration of response subsequent to active study phase (that is, how long any observed response is sustained). The main secondary outcome measure for the study is the level of depressive symptoms immediately post-treatment. This outcome is measured using the 10-item Montgomery Asberg Depression Rating Scale (MADRS), a standard clinician-administered measure of depressive symptoms with good reliability (Cronbach alpha, 0.85) and validity (coefficient, 0.314) in clinical populations [54, 55]. The MADRS has good responsiveness to the effect of antidepressant treatments and has been used in previous tDCS trials. The items are rated on a seven-point (0 to 6) scale (score range 0 to 60). To generate preliminary efficacy data that can be used to inform sample size calculations of the subsequent treatment trial, we will compare MADRS scores immediately after the active study phase between tDCS intervention and sham comparison groups, accounting for baseline MADRS scores in the analyses. We also measure depressive symptoms using the Edinburgh Postnatal Depression Scale (EPDS), a self-report depression screening measure that has been validated for use in pregnancy with a pooled internal consistency of 0.73 to 0.87 and test-retest reliability of 0.53 to 0.74 [56, 57]. Women with EPDS scores greater than 12 have ten times the likelihood of having diagnosis of major depressive disorder than women with EPDS less than 12 [58]. Although the scale itself is not diagnostic of major depressive disorder and is not traditionally used as a primary outcome measure in depression treatment studies, it has a better ability to detect women with depression in the perinatal period than traditional depression measures because of the increased weight given to anxiety symptoms that appear to be more common in perinatal than in nonperinatal depression. Additional maternal symptom measurements include the Pregnancy Experience Scale, as this has been validated against physiological measures in the developing fetus [59]. We will measure anxious symptoms using the State-Trait Anxiety Inventory, a self-report anxiety screening measure that has shown good discriminative validity in perinatal populations for the identification of anxiety disorders [60]. We also inquire about concurrent psychosocial or psychiatric health services to which participants may have been exposed (for example, psychotherapy, public health nursing, postpartum antidepressant use) as these exposures have the potential to confound treatment or safety effects if unbalanced between groups (see Table 1).

### Statistical methods

We will use descriptive methods to estimate feasibility and compliance for the number of tDCS intervention and sham sessions, the length of time each session lasted, follow-up rates at the various intervals, recruitment rate, rates of nonparticipation, and so on. We will calculate acceptability using Likert-type scale responses of the mothers’ satisfaction with the procedure and reported side effects, pregnancy, neonatal and child outcomes. Quantitative continuous measures will be compared between intervention and sham groups using *t* tests for normally distributed continuous variables, using chi-square tests of association for quantitative dichotomous measures or other nonparametric tests where appropriate. To compare depressive symptoms between depressed women receiving the tDCS intervention to depressed women receiving the control after 3 weeks of treatment analyses, we will use the intention-to-treat principle. Missing data points will be excluded from the analysis, and individuals with fewer than two depression measurements will be treated as lost to follow-up. An on-treatment analysis will also be performed as a sensitivity analysis (to determine the best possible performance of tDCS). Means of the MADRS scores of the intervention and control groups at the primary endpoint will be compared using a one-way analysis of covariance (ANCOVA) model, where the covariate will be the baseline MADRS score. This effect size will be used to generate the sample size needed for the future multicentre trial.

### Sample size

A review by Hertzog suggests a range of 20 to 40 participants to allow for sufficient variability in acceptability assessment of an intervention [61]. The referral sites receive approximately 100 referrals per month for antenatal depression combined, with approximately 20 being for moderate to severe depression in pregnancy. Based on recruitment for a previous study focused on women faced with decisions regarding antidepressant drug use in pregnancy [62], we estimate that ten patients (50%) will be ineligible because they accept antidepressant drug treatment, that four will be ineligible for other reasons and that three of the remaining six (50%) will consent to participate in the pilot study. Therefore, recruitment of 36 women could be achieved within 1 year. For women in the moderate depression range (that is, with a MADRS score of 16), this would allow us to detect a 30% difference in depressive symptom scores (clinically significant) between intervention and control participants with a probability (power) of 0.9 (alpha = 0.05).

### Safety monitoring

The study investigator team is well-positioned to assess and monitor safety in this pilot study given its collective expertise in brain stimulation, perinatal mental health, and obstetrics and gynecology. Safety and adverse events are assessed daily during the active phase and at all follow-up time points and are routinely reviewed by the principal investigator. An obstetrician co-investigator (blind to group allocation), reviews all fetal heart rate tracings after all routine tDCS and sham sessions, and is available (or has support) should any concerns arise during the sessions themselves. Urgent psychiatric issues of concern expressed to the obstetrical research nurse or the research coordinator would be: suicidal ideation, homicidal ideation, concern over harm to children or evidence of psychotic symptoms, or could include any other psychiatric concerns as per the judgement of the obstetrical research nurse or research coordinator. Urgent medical concerns expressed to the obstetrical research nurse or research coordinator would be considered to be any acute medical symptoms reported by the patient or observed by the study staff, as well as any concerns about fetal wellbeing expressed by the patient. In this case, the obstetrical research nurse or research coordinator would stop the session (if this occurs during treatment), keep the participant in the office (or on the telephone) and contact the study’s principal investigator (psychiatrist) for direction. The study’s principal investigator would assess the patient immediately, with input from the study obstetricians and maternal-fetal medicine specialists, as required. If no urgent medical or psychiatric concerns are identified based on this clinical assessment, the participant is directed back to her psychiatric or antenatal care provider. If necessary, the participant is directed to emergency services. If contact with participant is lost prior to assessment (that is, if adverse events are reported by telephone), then emergency services are contacted. Adverse events are recorded and immediately reported (within 24 hours by telephone or fax) to the research ethics boards of both institutions for consideration of further action (that is, unblinding of intervention, subject withdrawal, termination of study). Participants receive care as appropriate for any harm that arises as a result of study participation.

### Dissemination plan

The nature of this research is to determine whether it is feasible to conduct a large multisite RCT to evaluate the treatment effect of tDCS, a novel nondrug approach in women with moderate to severe depression in pregnancy. As such, the goals of the dissemination plan relevant to this pilot study are to: (1) inform future research in terms of providing evidence to proceed to a larger multicentre study of the efficacy of tDCS in treating pregnant women with depression; and (2) generate awareness and interest in both the research community and in the public about the potential of this treatment for preventing adverse child and maternal outcomes. This is highly important at this early stage in development, because once efficacy and safety are established, the long-term dissemination plan will be to generate action in practice change, promoting broad treatment uptake. With such low acceptability rates for treatment of depression in pregnancy, it is essential to involve practitioners and stakeholders throughout the initiation, conduct and outcome stages. This will help confront barriers to treatment acceptability (among patients and practitioners) by ensuring that clear, unbiased information is passed to potential treatment users so that they can make effective and timely decisions about treatment. To meet the first goal of this dissemination plan, potential partners for the planned multicentre trial require engagement. Our multidisciplinary team has connections with centres where women are treated for depression in pregnancy throughout Canada, the United States and internationally. Our team also has connections with clinical brain stimulation programmes at many of these sites, and can provide training in the use of the tDCS procedure. To meet the second goal of the dissemination plan, practitioners, the public, stakeholder groups and policy makers are target audiences. To engage our partners in the design and development of the multicentre trial, we will hold a trial planning meeting in the last stages of the current project (as long as our criteria for feasibility are met). We will also present the results of the pilot study in at least one national and one international conference in psychiatry or brain stimulation and submit results for peer-reviewed publication to promote the legitimacy of our findings. To communicate the findings to the broader community of providers, policy makers, women and their families in an encouraging and nonstigmatized manner, so that we can promote interest in participation in the larger treatment trial and later uptake of this treatment method, we will focus on generating awareness of the results of the pilot trial. We will disseminate the findings of our pilot study (after peer review) through our hospital websites and to our community stakeholders through small group presentations to the stakeholders at each of these agencies. We consider these presentations to be integrated knowledge transfer as they will be bidirectional in nature; they will help to inform us about patient needs and perceptions, while at the same time serving as mechanisms to disseminate information and generate interest and awareness of the potential of tDCS treatment.

## Discussion

Depression complicates up to 10% of pregnancies, more than gestational diabetes, hypertension or pre-eclampsia [1]. No standard treatment option comes without potential risks for mother or fetus. Transcranial direct current stimulation is a novel therapeutic approach to the treatment of depression during pregnancy. It has the potential to introduce a safe, effective, low-cost and accessible method for treatment of depression during pregnancy. The protocol described in this paper is a pilot RCT designed to guide the development of a larger multicentre study to definitively determine the safety and efficacy of tDCS as a treatment option for depression during pregnancy.

The strengths of our protocol include: (1) the use of a novel treatment option for depression during pregnancy that has not been previously described in this population; (2) robust fetal monitoring methods, to ensure the safety of both the mother and the unborn child, as well as to provide reassurance to the mother during treatment sessions; (3) extensive follow-up, to monitor the progress of the pregnancy, depressive symptoms and other health service use indicators that may influence outcomes; (4) study oversight by a strong team with a diverse range of expertise and training in the fields of reproductive and perinatal mental health, brain stimulation, obstetrical medicine and developmental neuroscience; and (5) a comprehensive dissemination plan to ensure adequate uptake of the knowledge that this study will generate. There are some limitations to the pilot study methodology, however. First, recruitment is limited to women seen at our specialty perinatal mental health clinics, and thus the recruitment and retention rates may not be applicable to centres with more general psychiatric or general obstetrical populations. That being said, our centres are likely to have the highest proportion of potentially eligible patients, making this an ideal setting for a pilot study. Second, we restricted our sample to women between 14 and 32 weeks gestation to increase the likelihood that women in our small planned sample would remain pregnant through active treatment. The disadvantage of this is that our study results are not necessarily applicable to women in early or late pregnancy, and that this will need to be addressed in future research. Finally, because this is a pilot study, our results will not support definitive conclusions on efficacy or safety of tDCS during pregnancy. In fact, they will help guide the development of a larger study to focus on these outcomes.

Some of the challenges of developing this protocol and the creative solutions to these challenges are worthy of discussion. Research has demonstrated that pregnant women are very hesitant to undergo any medical treatment during pregnancy (and providers are often reluctant to recommend treatments), out of concern for the safety of the fetus. Although tDCS represents no theoretical risk to the fetus, it has not been evaluated in pregnant patients. Therefore, we anticipated our main challenges to be with recruitment. To assist with this, we focused on both participants and providers. To assist patients in decision making, we allowed for the inclusion of partners in the informed consent process. Our rigorous fetal monitoring protocols were put in place not only to provide safety data relevant to the study, but also to provide reassurance to participants over the safety of the fetus during the procedure itself, possibly increasing the likelihood of participation. To engage providers, we presented on depression in pregnancy and on tDCS specifically to both the mental health and obstetrical departments in the respective institutions to ensure that a wide range of providers seeing these patients had a good understanding of the reasons for treating depression and of the proposed treatment with tDCS. These presentations led us to develop, at the suggestion of the practitioners, an information sheet for practitioners that could help guide their discussions. These interactive multidisciplinary presentations helped us to develop the content of the information sheets. For example, obstetrical providers indicated that their comfort with the tDCS procedures was increased when they considered the similarities to transcutaneous electrical nerve stimulation (TENS), a procedure used to relieve pain during labour and delivery. The strength of the TENS current is approximately 100 mA (50 to 100× the strength of tDCS), it is delivered directly to the abdomen, and many years of data indicate that it can be applied in pregnant populations with no ill effects [63]. We anticipate that education and support of the providers will aid in comfort of patients considering participation in the study.

In summary, this protocol was developed through strong collaborations between psychiatric and obstetrical service delivery providers and research practices. This includes recognition that provision of appropriate mental health care requires dialogue between all providers and care systems involved, particularly during vulnerable periods of time, such as during a pregnancy. This pilot RCT will allow us to assess the feasibility of a trial protocol for administering tDCS among pregnant women with moderate to severe depression. It will help guide the development of a larger, multicentre RCT to assess tDCS in this population that is in great need of a safe and effective treatment for depression.

### Trial status

Enrolment for this study began on 2 July 2014. At the time of submission, we have enrolled 0 participants.

## Abbreviations

ANCOVA: analysis of covariance
DSM-5: Diagnostic and Statistical Manual, fifth revision
EPDS: Edinburgh Postnatal Depression Scale
MADRS: Montgomery Asberg Depression Rating Scale
RCT: randomized control trial
RID: randomization identification number
tDCS: transcranial direct current stimulation
TENS: transcutaneous electrical nerve stimulation.
